# Improvement in Physical Function Associated With Burosumab in a Patient With X-linked Hypophosphatemia: A Case Report

**DOI:** 10.7759/cureus.90480

**Published:** 2025-08-19

**Authors:** Masato Fukushima, Ryuki Hashida, Hiroo Matsuse, Koji Hiraoka

**Affiliations:** 1 Division of Rehabilitation, Kurume University Hospital, Kurume, JPN; 2 Department of Orthopaedics Surgery, Kurume University School of Medicine, Kurume, JPN

**Keywords:** burosumab, fibroblast growth factor 23, monoclonal antibody, physical function, x-linked hypophosphatemic rickets/osteomalacia

## Abstract

Burosumab, a human immunoglobulin G1 monoclonal antibody that targets fibroblast growth factor 23 (FGF23), was developed to treat X-linked hypophosphatemic rickets/osteomalacia (XLH). By binding to FGF23, burosumab promotes the reabsorption of phosphorus from the kidney, increases the production of active vitamin D, and promotes the intestinal absorption of phosphorus and calcium. Herein, we report a one-year evaluation of physical function in adult patients with XLH who were treated using burosumab. A 40-year-old male patient with a history of lower limb varus deformity was diagnosed with vitamin D-resistant rickets in early childhood. Around October 2021, the patient began experiencing pain in both lower limbs when ascending and descending stairs at his place of work, making it difficult for him to perform his duties. We prescribed subcutaneous burosumab (70 mg) in December 2022. Twelve weeks after starting this treatment, his 10-meter walking speed and Time Up-and-Go Test results showed significant improvement. We therefore concluded that burosumab treatment may improve physical function in adult patients with XLH.

## Introduction

Fibroblast growth factor 23 (FGF23) was first reported as the causative gene of autosomal dominant hypophosphatemic rickets in 2000 [1]. FGF23 is a hormone produced by bone cells that plays a central role in regulating blood phosphorus levels by acting on the kidneys and intestinal tract. Excessive FGF23 production lowers serum phosphorus levels by suppressing phosphorus reabsorption in the renal proximal tubules, as well as phosphorus absorption from the intestinal tract, through a decrease in 1,25(OH)_2_D concentration. This can result in the development of hypophosphatemic rickets/osteomalacia (such as in patients with X-linked hypophosphatemic rickets/osteomalacia {XLH}), as well as tumor-induced osteomalacia (TIO) [2].

These diseases have been conventionally treated with phosphorus and active vitamin D preparations; however, several adverse events have been reported, including renal and urinary tract stones, gastrointestinal symptoms such as diarrhea, and secondary-to-tertiary hyperparathyroidism [1]. Burosumab, a human immunoglobulin G1 monoclonal antibody that targets FGF23, was recently developed to treat these conditions. It became available for clinical use in 2018 and was approved for insurance coverage in Japan in 2019 for the treatment of FGF23-related hypophosphatemic rickets and osteomalacia [3]. By binding to FGF23, burosumab promotes phosphorus reabsorption from the kidneys, increases active vitamin D production, and enhances the intestinal absorption of phosphorus and calcium [3]. Clinical trials in adult patients with XLH have reported significant increases in serum phosphorus levels after 24 weeks in patients treated with burosumab compared to those treated with a placebo [4]. Clinical trials focused on physical function have also reported improvements in patients’ Western Ontario and McMaster Universities Osteoarthritis Index scores following treatment with burosumab [3].

To date, there have been several reports discussing clinical results following treatment with burosumab, but few have examined changes in physical function over time in adult patients with XLH who received long-term burosumab treatment. Herein, we discuss the relationship between serum phosphorus concentration and various patient parameters, as analyzed via blood tests, physical function, and quality of life assessments, over an extended period following the administration of burosumab.

## Case presentation

Our patient was a male in his 40s. He was 155.0 cm tall, weighed 65 kg, and had a body mass index of 27.06 kg/m². At the age of three years, he was diagnosed with vitamin D-resistant rickets after visiting our hospital because of an internal deformity in his lower limbs. He continued to visit an outpatient clinic for this condition until 2009, after which he managed it through regular checkups with his local doctor. However, in October 2021, he began experiencing pain in both his lower limbs when ascending and descending stairs at his workplace, which made it difficult for him to perform his duties there. Although this task became easier for him following treatment with fosulibone tablets, the patient still had residual weakness in his lower extremities and stiffness during the initial phases of walking. His chief complaints were difficulty walking long distances continuously and using stairs. The patient was started on subcutaneous burosumab injections (70 mg) in December 2022. To evaluate their effects, physical function assessments were conducted over the following year. Informed consent was obtained from the patient for the publication of this study and accompanying images.

Burosumab was administered subcutaneously every four weeks at a dose of 70 mg. The physical function evaluation period spanned 12 injections and was performed before each one. The first, second, third, fourth, fifth, sixth, eighth, 10th, and 12th evaluation periods were each conducted nine times. The assessments included blood chemistry analysis, grip strength, 10-meter walking speed, Time Up-and-Go Test (TUG), time to stand on one leg, knee extension strength, chair-and-standing 5 test (CS5), 2-step test, 25-question Geriatric Locomotive Function Scale (GLFS-25), and Barthel index. These items were chosen because they are associated with sarcopenia and locomotive syndrome.

The results of the patient’s initial and final evaluations are presented in Table 1. His serum phosphorus level initially decreased to 1.5 mg/dL. The patient remained independent in terms of performing activities of daily living, but his overall physical function exhibited a significant decline. He had a GLFS-25 score of 23, indicating locomotive syndrome stage 2. However, his physical function improved significantly following the burosumab treatment. The patient’s serum phosphorus levels rose from an initial 1.5 mg/dL to a peak of 2.8 mg/dL (Table 1).

**Table 1:** Patient’s characteristics in the initial and final evaluation. P: phosphorus; ADL: activities of daily living; ALP: alkaline phosphatase

Item		Reference range	Initial evaluation	Final evaluation
Blood collection data	P (mg/dL)	2.7~4.6	1.5	2.5
	Ca (mg/dL)	8.8~10.1	9.2	9.5
	ALP (U/L)	106~322	242	198
Physical function assessment	Grip strength: dominant hand (kg)	≥26	21.5	34.6
	10-meter walking speed (m/s)	≥1.4	1.45	1.9
	Time Up-and-Go Test (s)	<13.5	9.10	6.72
	Knee extensor strength: dominant leg (N/kg)	≥1.0	1.45	6.42
	Chair-and-standing 5 test	<12	18.21	9.46
Locomotive syndrome assessment	Two-step test	≥1.3	0.98	1.21
	Stand-up test	One leg 40 cm possible	Both legs 20 cm possible, one leg 40 cm not possible	Both legs 20 cm possible, one leg 30 cm possible
	25-Question Geriatric Locomotive Function Scale (points)	<16	23	6
ADL assessment	Barthel Index (points)	100	100	100

**Figure 1:**
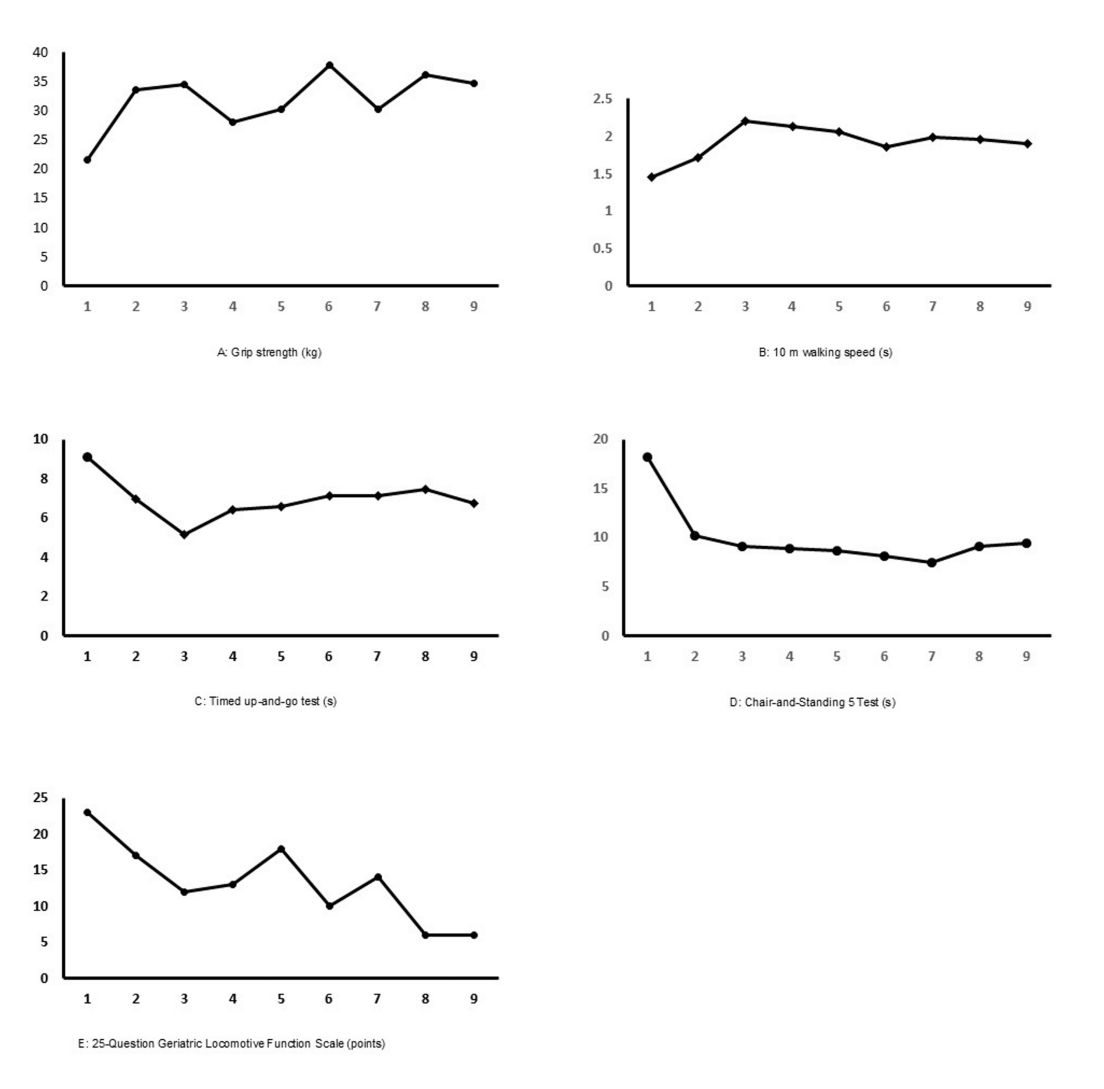
Changes in physical function with burosumab administration. The vertical axis shows each assessment, and the horizontal axis shows the injection times of burosumab.

His grip strength improved by a maximum of ~1.7-fold compared to the first assessment (from 21.5 kg to 34.6 kg at the final assessment) (Figure 1A); his 10-meter walking speed and TUG improved by ~1.5-fold each (10-meter walking speed: from 1.45 m/s to 1.9 m/s by the final assessment; Figure 1B) (TUG: from 9.1 s to 6.72 s by the final assessment; Figure 1C). However, no further improvements were observed after the third dose. His CS5 time also showed a significant improvement from the first assessment (from 18.21 s to 9.46 s by the final assessment) (Figure 1D). However, this improvement remained similarly unchanged after the second dose. The patient had an initial score of 23 and locomotive syndrome stage 2 on the GLFS-25, but eventually improved to locomotive syndrome stage 1 and a score of 6 points (Table 1, Figure 1E).

## Discussion

In this study, a patient with XLH was treated with burosumab for one year, during which changes in serum phosphorus levels and physical function were monitored. Our patient’s initial serum phosphorus level was critical at 1.5 mg/dL. Phosphorus is involved in adenosine triphosphate (ATP) synthesis, so we hypothesized that our patient likely had decreased intracellular ATP production. Patients in states of hypophosphatemia often experience difficulty with sustained muscle contraction due to decreased ATP synthesis in skeletal muscles [5]. This manifestation is consistent with our patient‘s chief complaints of difficulty walking long distances continuously and ascending and descending stairs. We also considered that the patient likely had decreased muscle strength, including grip strength, during our initial physical therapy evaluation, thus posing a risk of locomotive syndrome. In XLH, musculoskeletal symptoms such as muscle weakness, increased fatigability, deconditioning, and pain can cause motor impairment, impaired daily functioning, and decreased overall quality of life [6]. Veilleux et al. reported no change in muscle cross-sectional areas in a cohort of patients with XLH, although their muscle strength was significantly lower than that of healthy controls. This suggests that the symptoms of XLH are likely not caused by disuse-related muscle atrophy, muscle weakness, or neurogenic muscle weakness seen in normal practice [7]. Thus, we assume that the administration of burosumab in this case facilitated sustained muscle contraction by increasing ATP production in response to higher serum phosphorus levels. However, we did not directly assess ATP production in this study; the impact of changes in serum phosphorus on ATP production is a topic for future study. Moreover, physical functions such as prolonged walking ability should have been assessed using exercise tolerance tests, including the 6-minute walk and the 30-second sit-to-stand test.

Burosumab successfully maintained serum phosphorus levels within the normal range throughout the one-year treatment period, fulfilling its intended pharmacological role. A significant improvement in physical function was observed within three months of the first dose, but no further substantial changes occurred thereafter; rather, the improvement was sustained. This suggests that burosumab may correct the hypophosphatemic state, thereby facilitating improvements in physical function. In this case, the improvement in physical function paralleled the normalization of serum phosphorus and was maintained with continued treatment. Therefore, long-term administration of burosumab may contribute to maintaining both serum phosphorus levels and physical function. A comprehensive treatment strategy combining pharmacotherapy with physiotherapy may yield further benefits. Moreover, no serious adverse events, including those related to hormonal imbalance, were reported during the one-year treatment period, indicating a favorable safety profile.

Future prospects related to exercise-based therapy for improving physical and bone function in patients with XLH should continue to be investigated as well. Our patient initially presented with decreased muscle strength and balance compared to healthy adults of the same age. The goal of physical therapy in patients with XLH is to alleviate pain and improve physical function in order to reduce XLH-related disability; however, there have been no disease-specific recommendations made regarding physical therapy for patients with XLH to date. Therefore, rehabilitation treatment is typically implemented based on standard-practice physical therapy recommendations for knee or hip osteoarthritis. Future recommendations for exercise therapy specifically tailored to patients with XLH are therefore eagerly anticipated.

## Conclusions

We have observed patients with X-linked hypophosphatemia (XLH) treated with burosumab. In the present case, improvement in physical function paralleled an increase in serum phosphorus levels and was sustained with continued treatment. Therefore, long-term administration of burosumab may help maintain both serum phosphorus levels and physical function in patients with XLH.
